# Inhibition of HIF-1*α* Affects Autophagy Mediated Glycosylation in Oral Squamous Cell Carcinoma Cells

**DOI:** 10.1155/2015/239479

**Published:** 2015-11-11

**Authors:** Yi-Ning Li, Ji-An Hu, Hui-Ming Wang

**Affiliations:** ^1^Department of Pathology, Stomatology Hospital, College of Medicine, Zhejiang University, Hangzhou, Zhejiang 310006, China; ^2^Department of Oral and Maxillofacial Surgery, Stomatology Hospital, College of Medicine, Zhejiang University, Hangzhou, Zhejiang 310006, China

## Abstract

*Purpose*. To validate the function of autophagy with the regulation of hypoxia inhibitor-induced glycosylation in oral squamous cell carcinoma cell. *Methods*. Human Tca8113 cell line was used to detect autophagy and glycosylation related protein expression by western blotting and immunofluorescence with HIF-1*α* inhibitor. Short interfering RNA (siRNA) transfection blocked human ATG12 and ATG1. *Results*. HIF-1*α* inhibitor PX-478 reduced the amount of LC3-II and LC3-I in Tca8113 cells. PX-478 decreased the expression of O-GlcNAc and OGT and increased OGA expression. The tendency of O-GlcNAc showed a similar pattern to OGT. PX-478 gradually decreased OGT expression in Tca8113 cells. Protein level of O-GlcNAc and OGT increased in ATG12 and ATG1 depletion. The expression of OGT decreased at first and then rose slowly with the treatment of Atg12 and Atg1 siRNA and PX-478 fluctuant. Autophagy affected the stability of OGT when HIF-1*α* signaling was blocked. *Conclusions*. Autophagy reduced by hypoxic stress inhibited. HIF-1*α* inhibitor decreased glycosylation. OGT became unstable in the absence of autophagy when HIF-1*α* signaling was blocked.

## 1. Introduction

Autophagy's basic role in the turnover of proteins and organelles, digesting cellular contents to provide cellular energy and building blocks for biosynthesis, possesses multiple physiological and pathophysiological functions in the maintenance of cellular homeostasis. It has early been demonstrated to play a significant role in tumorigenesis, but whether it acts as a promoter or a suppressor during tumorigenesis seems to be context-specific [1].

Transcription factor hypoxia-inducible factor-1 (HIF-1) is the key regulator of the cellular response to hypoxia which is composed of an oxygen dependent subunit (HIF-1*α*) and a constitutively expressed nuclear subunit (HIF-1*β*) [2]. Cellular adaptation to hypoxia is critical to malignant tumor progression and survival [3]. Hypoxia is capable of rapidly inducing, via the hypoxia-inducible factor (HIF-1), a cell survival response engaging autophagy [4]. Downregulation of HIF-1*α* is capable of suppressing AC A549 cell growth, through the induction of apoptosis [5]. PX-478 (S-2-amino-3-[4′-N,N-bis(2-chloroethyl)amino]phenyl propionic acid N-oxide dihydrochloride) is an inhibitor of constitutive and HIF-1*α* levels. HIF-1*α* subunit regulates the activity of HIF-1. PX-478 inhibits HIF-1*α* at protein levels and transactivation in a variety of cancer cell lines by decreasing levels of HIF-1*α* mRNA and inhibiting translation [6–8].

Glycosylation research is hot now, but the mechanism is not clear yet which may be caused by elevated levels of O-GlcNAcylation by O-linked *β*-N-acetylglucosamine (O-GlcNAc) or O-GlcNAc transferase (OGT). Literatures reported about some cancers indicated that glycosylation played an important role in the development, cell adhesion, and invasion of cancer, such as pancreatic cancer [9], breast tumors [10], and gastric cancer in patients [11]. Absent *α*-dystroglycan (*α*-DG) expression and like-acetylglucosaminyltransferase (LARGE) deregulation were closely associated with nodal metastasis of tongue cancer [12]. Recent research found that O-GlcNAcylation regulated cancer metabolism signaling via regulation of the HIF-1 pathway. HIF-1*α* was critical for OGT-mediated regulation of metabolic stress, as overexpression of stable HIF-1 rescues metabolic defects. Human breast cancers with high levels of HIF-1*α* contain elevated OGT, and lower OGA levels correlate independently with poor patient outcome [13].

To validate the putative participation of autophagy in the regulation of hypoxia inhibitor-induced glycosylation, we examined the effects of hypoxia on autophagic activity of in oral squamous cell carcinoma cells. Then we investigated the HIF-1's function in glycosylation. Finally, we explored potential mechanisms of OGT with both blocked HIF-1 and autophagy.

## 2. Materials and Methods

### 2.1. Cell Culture

Human Tca8113 cell lines were obtained from ATCC (BioHermes, Shanghai, China). MBM was cultured in *α*-modified Eagle's medium (*α*-MEM; GIBCO-BRL) with 10% fetal bovine serum (FBS; GIBCO-BRL) and was maintained under a humidified atmosphere and 5% carbon dioxide at 37°C.

### 2.2. Western Blotting, Immunofluorescence (IF) Antibodies, and Reagents

The used antibodies and reagents were as follows: O-GlcNAc mouse monoclonal (Thermo, USA), OGT rabbit polyclonal (Abcam, USA), OGA rabbit monoclonal (Abcam, USA), LC3-II rabbit polyclonal (Sigma, USA), and LC3-I rabbit polyclonal (Sigma, USA); GAPDH (Thermo, USA), double-stranded siRNA targeting human ATG12 and ATG1 (Invitrogen, USA); PX-478 (ProlX, Tucson, USA).

### 2.3. Short Interfering RNA (siRNA) Transfection

Double-stranded siRNA targeting human ATG12 and ATG1 (purchased from Invitrogen) were administered simultaneously (30 nM each) to Tca8113 cells in Lipofectamine RNAiMAX reagent. In all experiments, scrambled siRNA served as a control. Tca8113 cells were analyzed 48 h after transfection. Protein knockdown was assessed by western blot analysis.

### 2.4. Statistical Analysis

Experimental data are reported as mean ± SD of triplicate independent samples. All experiments were performed in triplicate on three independent occasions. Data were analyzed with two-tailed Student's *t*-test. *p* values < 0.05 were considered significant.

## 3. Results

### 3.1. HIF-1*α* Inhibitor PX-478 Reduces Cellular Autophagy in Tca8113 Cells

**Figure 1:**
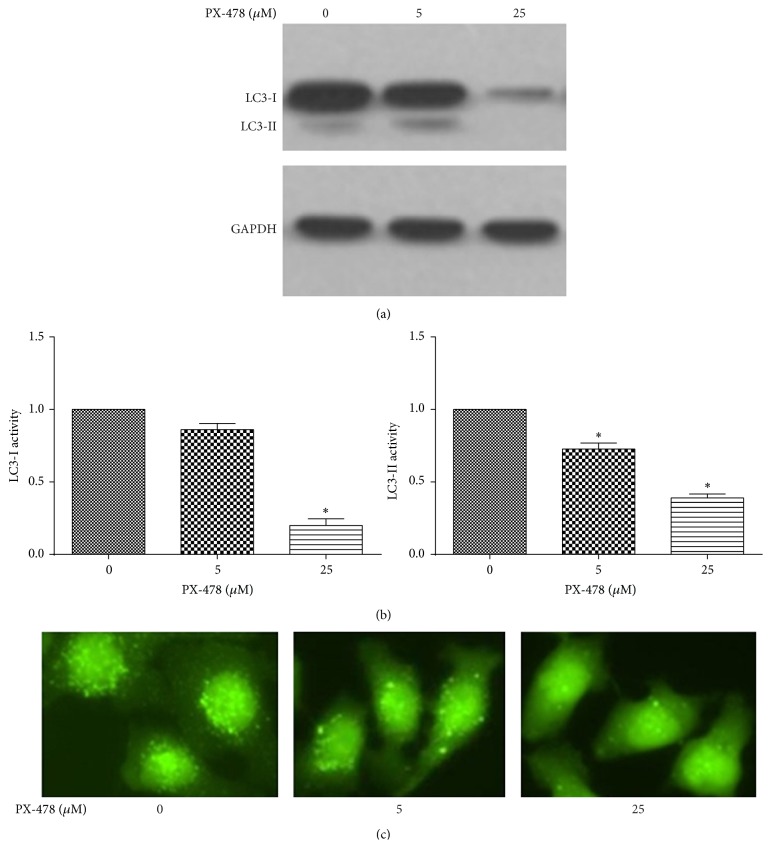
HIF-1*α* inhibitor PX-478 reduces cellular autophagy in Tca8113 cells. (a) Representative western blots showing LC3-II and LC3-I in PX-478 treated Tca8113 cells. Tca8113 cells were treated with 0, 5, and 25 *μ*M PX-478 for 24 h. (b) Quantification of the amount of LC3-II and LC3-I (^*∗*^ *p* < 0.05, Student's *t*-test). The data are presented as mean ± SD for three independent experiments. (c) Autophagosome could be seen to have variation with 0, 5, and 25 *μ*M PX-478 treated cells (400x).

HIF-1*α* could regulate autophagy and cell proliferation. MAP-LC3 is a major constituent of the autophagosome. During autophagy, the cytoplasmic form (LC3-I) is processed and recruited to the autophagosomes. The hallmark of autophagic activation is the formation of cellular autophagosome punctae containing LC3-II, while autophagic activity is measured biochemically as the amount of LC3-II that accumulates in the absence or presence of lysosomal activity [14]. Induction of autophagy induces LC3-I conversion, producing lapidated LC3-II by action of Atg12-Atg5-Atg16L complex [15]. When autophagy was degraded by treatment of HIF-1*α* inhibitor PX-478 (0, 5, and 25 *μ*M), the amount of LC3-II and LC3-I decreased (Figures 1(a) and 1(b)). Autophagosome could be seen decreased with the dose increase of PX-478 (Figure 1(c)). These results suggested that HIF-1*α* blocking could reduce autophagy.

### 3.2. PX-478 Affected Glycosylation by Decreasing O-GlcNAc and OGT and Increasing OGA Expression

**Figure 2:**
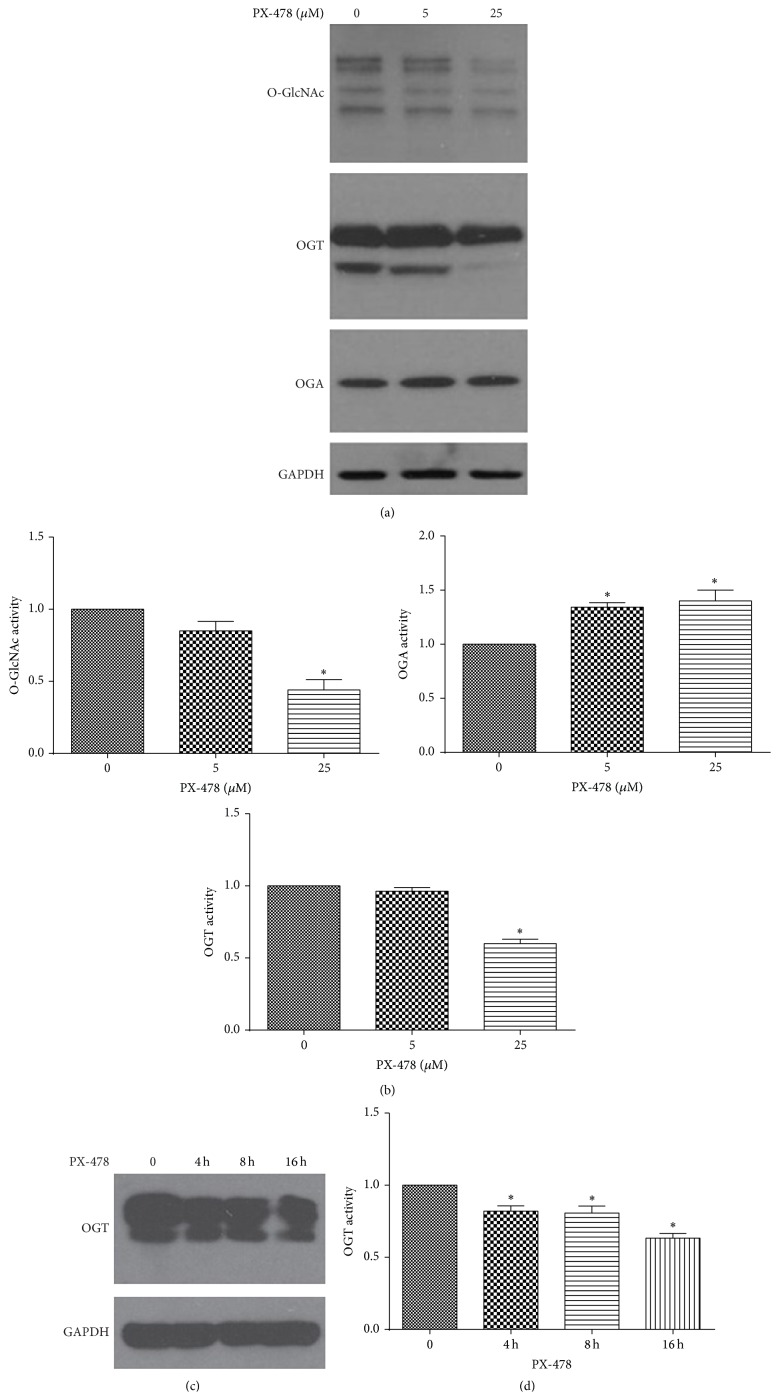
Protein expression of O-GlcNAc, OGT, and OGA changes in HIF-1*α* inhibition. (a) Lysates from Tca8113 cells treated with HIF-1*α* inhibitors were analyzed by western blotting using O-GlcNAc, OGA, OGT, and GAPDH antibodies. (b) Quantification of O-GlcNAc, OGT, and OGA (^*∗*^ *p* < 0.05, Student's *t*-test). The data are presented as mean ± SD for three independent experiments. (c) Western blots showing Tca8113 cells were treated with 25 *μ*M PX-478 for 0, 4, 6, and 8 h. (d) Quantification of the amount of OGT bands in (c) (^*∗*^ *p* < 0.05, Student's *t*-test).

To determine whether HIF-1*α* inhibition affected glycosylation modification in Tca8113 cells, we detected* O*-GlcNAcylation varied in PX-478 treated Tca8113 cells. Interestingly, total* O*-GlcNAcylation was decreased in HIF-1*α* inhibitor PX-478 Tca8113 cells. Then we detected OGT and O-GlcNAcase (OGA) protein expression due to the O-GlcNAc decreased in HIF-1*α* inhibition. OGT expression was decreased and OGA was increased under PX-478 treatment (Figures 2(a) and 2(b)). PX-478 affected protein expression of OGT and OGA in an opposite way. The tendency of O-GlcNAc showed a similar pattern to OGT.

We treated PX-478 with 25 *μ*M for 0, 4, 8, and 16 h in Tca8113 cells to know whether OGT variation occurs in short-term inhibition of HIF-1*α* or not. PX-478 treatment for 4–16 h gradually decreased OGT expression in Tca8113 cells (Figures 2(c) and 2(d)). This result implied that OGT decrease in HIF-1*α* inhibitor treatment for a time frame might be related to autophagic induction.

### 3.3. Atg12 siRNA and Atg1 siRNA Transfection Increase Glycosylation

**Figure 3:**
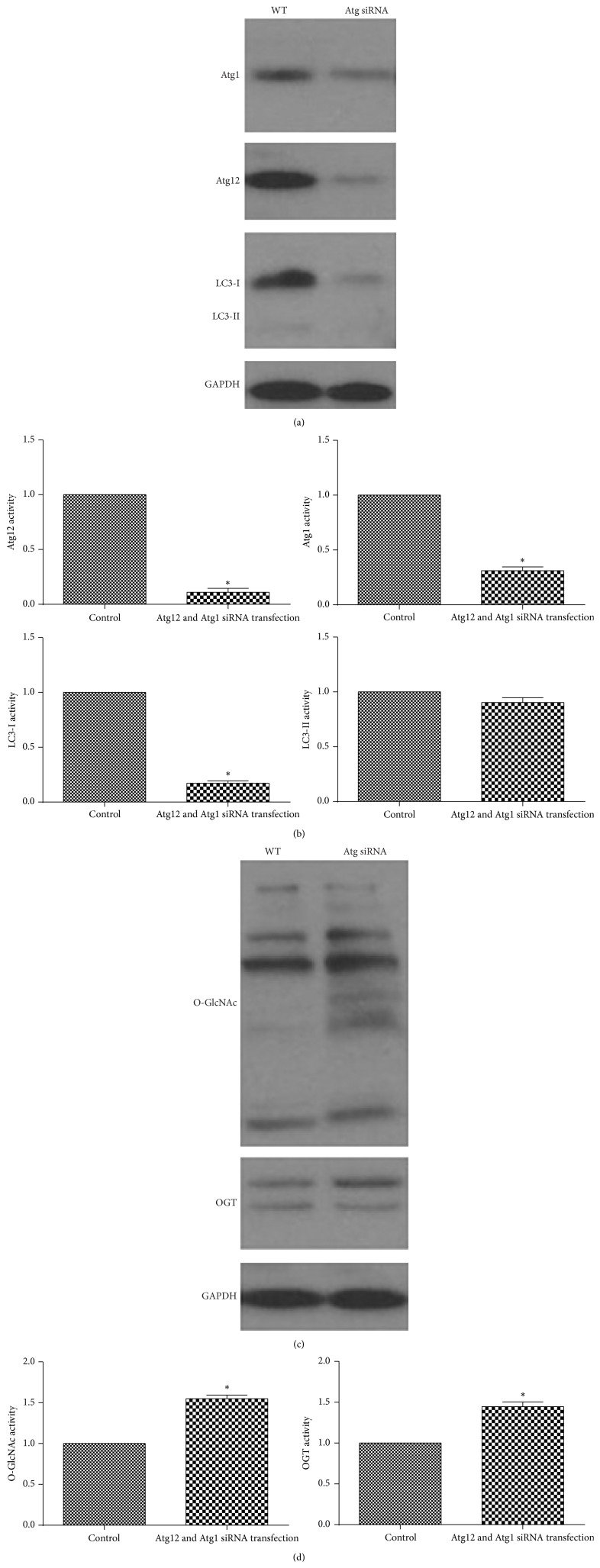
Atg12 siRNA and Atg1 siRNA transfection increase O-GlcNAc and OGT in Tca8113 cells. (a) After transfection with ATG12 and ATG1 siRNA for 48 h, the protein levels of ATG12, ATG1, LC3-II, and LC3-I were analyzed by western blotting. (b) Quantifications of protein amount in (a) separately (^*∗*^ *p* < 0.05, Student's *t*-test). (c) Lysates from Tca8113 cells transfected with ATG12 and Atg1 siRNA were analyzed by western blotting using O-GlcNAc, OGT, and GAPDH antibodies. (d) Quantification of protein amount in (c) (^*∗*^ *p* < 0.05, Student's *t*-test).

To study whether autophagy affects glycosylation variation, we used Atg12 siRNA and Atg1 siRNA to reduce formation of autophagosome. Conversion of LC3-I to LC3-II decreased in depletion of ATG12 and ATG1 (Figures 3(a) and 3(b)). Protein level of O-GlcNAc and OGT increased in ATG12 and ATG1 depletion (Figures 3(c) and 3(d)). Our result declared that inhibited autophagosome could induce accumulation of O-GlcNAc and OGT protein in Tca8113 cells.

### 3.4. Inhibition of Autophagy Restores Protein Level of O-GlcNAc and OGT under HIF-1*α* Inhibition

**Figure 4:**
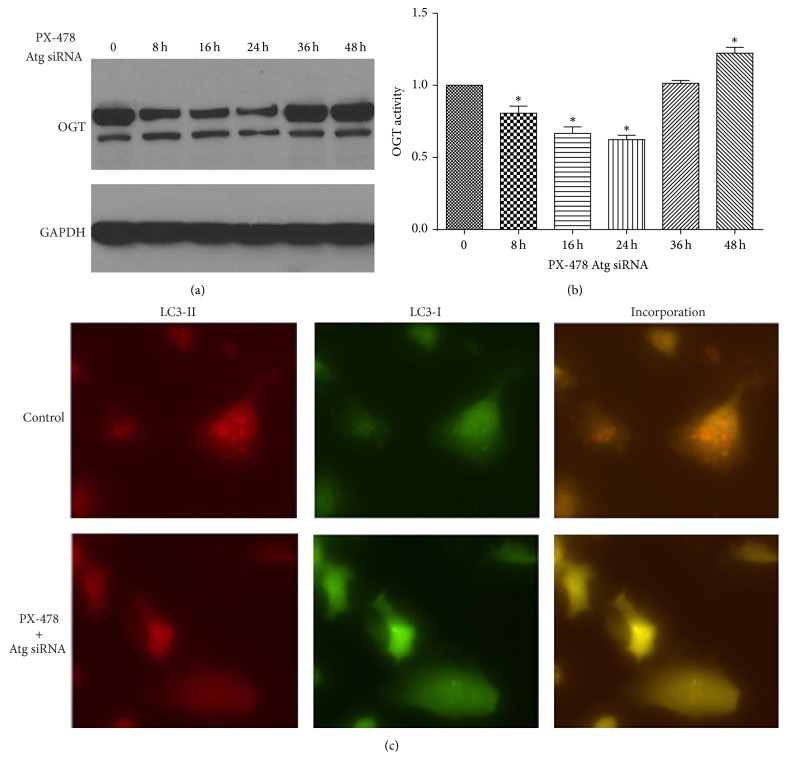
Atg12 and Atg1 siRNA restore protein level of O-GlcNAc and OGT under PX-478 treatment. (a) The expression of OGT in 0, 8, 16, 24, 36, and 48 h after treatment of Atg12 and Atg1 siRNA and PX-478. (b) Quantifications of protein amount in (a) (^*∗*^ *p* < 0.05, Student's *t*-test). (c) LC3-I and LC3-II expression in the 48 hours in Atg12 and Atg1 siRNA and PX-478 treated Tca8113 cells in immunofluorescence assay (400x).

To clarify the involvement of autophagy and HIF-1*α* in regulation of the glycosylation, we designed the OGT protein detected based on the autophagy and HIF-1*α* inhibited at the same time. The expression of O-GlcNAc was related to OGT, so we tested whether prohibition of Atg12 and Atg1 siRNA can restore the protein level of OGT after PX-478 treatment. The expression of OGT in 0, 8, 16, 24, 36, and 48 h after treatment of Atg12 and Atg1 siRNA and PX-478 fluctuant decreased at the first 24 hours and then rose slowly (Figures 4(a) and 4(b)). Therefore, we considered that protein instability of OGT was mainly due to the induction of autophagy at last, partially by the inhibition of HIF-1*α* at the beginning period. LC3-I and LC3-II were totally inhibited in the 48 hours in immunofluorescence assay (Figure 4(c)). This implied that autophagy still affects the stability of OGT when HIF-1*α* signaling was blocked.

## 4. Discussion

### 4.1. HIF-1*α* Inhibitor Reduces Cellular Autophagy

In this study we were able to link hypoxia and autophagy in Tca8113 tumor cell lines. We found that when HIF-1*α* was blocked, autophagy reduced with autophagosome and LC3-II/LC3-I decreased. Our result showed that hypoxia positively related to the autophagy in tumor cells. Also, Zhao et al. [16] found that knockdown HIF-1*α* abrogated hypoxia-induced autophagy activation in osteoclast cells. The invasion and vascular remodeling under hypoxia were significantly reduced in autophagy-deficient cells [17].

There are many pathways involved in HIF-1*α* affected autophagy. The resistance against cell death observed under hypoxia can be explained by a more effective autophagic flow activated via the classical mTOR pathway [18]. HIF-1*α* binds to effectors of chaperone-mediated autophagy (CMA) and is targeted for lysosomal degradation [19].

### 4.2. HIF-1*α* Inhibitor Decreases Glycosylation

HIF-1 promotes glycogenesis, or increased production of glycogen from glucose [20]. Block OGT for inhibition of Sp1 O-GlcNAcylation and Sp1 siRNA significantly reduced GlcN-induced HIF-1*α* protein expression under hypoxia [21]. Administration of the oxidative stress inhibitors reversed both proteasome activation and OGT degradation [22].

To study the expression of O-GlcNAc, OGT, and OGA affected by the HIF-1*α* inhibition in Tca8113 cells, OGT expression was decreased and OGA was increased under PX-478 treatment. These data consisted with the O-GlcNAc modification on proteins expression declined under HIF-1*α* inhibition, indicating that decreasing of OGT for a time frame with metabolism variation may relate to cell death caused by autophage. Our observations consisted with previous findings that Sp1 was being involved in the basal transcriptional activation of HIF-1*α* in breast cancer cells [23]. Tumor cells exhibited significant differences from normal cells in their metabolism. The interplay between the cellular metabolic network, oncogenes, tumor suppressors, hypoxia, autophagy, and biosynthetic anabolism is a hot topic [24].

### 4.3. HIF-1*α* Inhibitor Block Autophagy Mediated Glycosylation Increasing

In the present study, we investigated glycosylation variation in Atg12 and Atg1 knockdown in Tca8113 cell lines. Our result declared that the inhibition of autophagy induced accumulation of glycosylation. Knockdown Atg normal mRNAs suppress the formation of autophagosome and showed accumulation of O-GlcNAc and OGT. Thus, we considered autophagy to be one of the fundamental degradation ways of OGT. To analyze whether the role of autophagy affecting the glycosylation in tongue squamous cell carcinoma is dependent on the variation of the hypoxia, we designed the OGT protein detected based on the autophagy knockdown while HIF-1*α* was inhibited. Interestingly, HIF-1*α* does not seem to play an important role in the autophagy on regulation of glycosylation in the long term although it has the critical effect on the expression of OGT. Our results indicated that OGT became unstable in the absence of autophagy when HIF-1*α* signaling was blocked. HIF-1*α* plays a main role at the early stage in the process of this dynamic change.

Hypoxia, autophagy, and glycosylation are all physiological conditions in tissues in vivo. They are all concerned the cells lifetime [25]. The relationship in hypoxia, autophagy, and glycosylation is complicated. Further functional experiments will be needed to support the direct roles of glycosylation mediated autophagy on HIF-1*α* at molecular and cellular level.
